# Melanoma cell migration is upregulated by tumour necrosis factor-*α* and suppressed by *α*-melanocyte-stimulating hormone

**DOI:** 10.1038/sj.bjc.6601698

**Published:** 2004-03-02

**Authors:** N Zhu, R Lalla, P Eves, T L H Brown, A King, E H Kemp, J W Haycock, S MacNeil

**Affiliations:** 1 Section of Human Metabolism, Division of Clinical Sciences (North), Northern General Hospital, Sheffield S5 7AU, UK; 2 Department of Engineering Materials, University of Sheffield, Sir Robert Hadfield Building, Mappin Street, Sheffield S5 7AU, UK; 3 Department of Reconstructive Burns and Plastic Surgery, Northern General Hospital Trust, Sheffield S5 7AU, UK; 4 Cardiovascular Research Group, Division of Clinical Sciences (North), Northern General Hospital, Sheffield S5 7AU, UK

**Keywords:** melanoma, *α*-MSH, inflammation, integrin, melanocortin

## Abstract

We reported recently that the inflammatory cytokine tumour necrosis factor *α* (TNF-*α*) can upregulate integrin expression, cell attachment and invasion of cells through fibronectin in a human melanoma cell line (HBL). Furthermore, the actions of TNF-*α* were suppressed by the addition of an anti-inflammatory peptide *α*-melanocyte-stimulating hormone (*α*-MSH). In the current study, we extend this work investigating to what extent TNF-*α* might stimulate melanoma invasion by promoting cell migration and whether *α*-MSH is also inhibitory. Two human melanoma cell lines were examined *in vitro* (HBL and C8161) using a scratch migration assay. Analysis using either time-lapse video microscopy or imaging software analysis of migrating ‘fronts’ of cells revealed that C8161 cells migrated more rapidly than HBL cells. However, when cells were stimulated with TNF-*α* both cell types responded with a significant increase in migration distance over a 16–26 h incubation time. *α*-Melanocyte-stimulating hormone had an inhibitory effect on TNF-*α*-stimulated migration for HBL cells, completely blocking migration at 10^−9^ M. In contrast, C8161 cells did not respond to *α*-MSH (as these cells have a loss-of-function melanocortin-1 receptor). However, stable transfection of C8161 cells with the wild-type melanocortin-1 receptor produced cells whose migration was significantly inhibited by *α*-MSH. In addition, the use of a neutralising antibody to the *β*_1_-integrin subunit significantly reduced migration in both cell types. This data therefore supports an inflammatory environment promoting melanoma cell migration, and in addition shows that *α*-MSH can inhibit inflammatory stimulated migration. The data also support a fundamental role of the *β*_1_-integrin receptor in melanoma cell migration.

Despite public education on the risk of sunshine and associated UV light exposure and the increasing number of dermatology screening clinics, the incidence of melanoma continues to rise (Oliveria *et al*, 2001; Lang, 2002). Although treatment of early stage tumours is largely successful, nodal metastasis is associated with 70% mortality after 10 years if multiple lymph nodes are involved (Soong *et al*, 1992). Of particular concern are clinical observations that following excision of a primary malignant melanoma reoccurrence can arise at the wound site after a number of months. In support of this observation, animal studies demonstrate that a wound-healing environment can enhance local tumour growth and accelerate the development of distant melanoma metastases (Bogden *et al*, 1997; Hofer *et al*, 1998).

One of the very first events in a normal healing wound is an acute inflammatory response. Inflammation is needed to combat possible infection but also to stimulate cellular proliferation necessary for tissue repair. However, it is possible that the same early inflammatory signals may also promote malignant melanoma invasion and metastasis. The strongest evidence implicating an inflammatory response in the development of metastases is from colorectal cancer, where anti-inflammatory drug use is associated with a lower incidence of tumour recurrence (Benamouzig *et al*, 2001). A study of oral indomethacin and ranitidine in patients with advanced melanoma reported a partial response in two out of 17 patients, with reduction of metastatic tumour mass (Mertens and Lohmann, 1996). Indomethacin has been found to inhibit metastases and influence the cytotoxic activity of natural killer cells both *in vitro* and in animal models (Bigda and Mysliwski, 1998), and also induces apoptosis in oesophageal carcinoma cells (Aggarwal *et al*, 2000) and prostatic carcinoma cell lines (Andrews *et al*, 2002). In support of these observations, salicylic acid (a closely related anti-inflammatory compound) is reported to inhibit breast cancer cell growth (Sotiriou *et al*, 1999).

In common with other malignancies, an inflammatory stress, such as sublethal laser radiation (Zhu *et al*, 1997,^1999^), can promote metastasis of melanoma (Giavazzi *et al*, 1990; Dekker *et al*, 1994; Link *et al*, 1999). Changes in the expression of cellular adhesion molecules (Hynes, 1992; Natali *et al*, 1993; Danen *et al*, 1995; Stuiver and O'Toole, 1995) and matrix metalloproteinase activity (Hojo *et al*, 2002) are strongly associated with an increase in melanoma phenotype and invasion. Of particular note is that integrin and adhesion molecule expression can be directly upregulated by proinflammatory cytokines within the local cellular environment (Creyghton *et al*, 1995).

In support of the above, we have recently shown that integrin expression, cell attachment and invasion through fibronectin are upregulated by the proinflammatory cytokine TNF-*α* and opposed by *α*-MSH (Zhu *et al*, 2002) in a human cutaneous melanoma cell line (HBL). We also found TNF-*α* to cause a modest increase in proteolytic activity, which could be blocked effectively using *α*_2_ macroglobulin (Katerinaki *et al*, 2003), but with no concomitant increase in matrix metalloproteinase 2 or 9 activation or expression. However, the above studies did not consider whether melanoma invasion through fibronectin was attributable specifically to an increase in cell migration or to an increase in invasion. The use of *in vitro* scratch assays has previously enabled study of cellular migration alone (e.g. inhibition of neuroblastoma migration by retinoic acid (Voigt and Zintl, 2003) and the role of *α*-2 integrin expression in ovarian carcinoma cell migration (Kokenyesi *et al*, 2003)). Therefore, in the present study, we have investigated whether the inflammatory cytokine TNF-*α* acts by promoting melanoma cell migration *in vitro*, using this approach. In addition, we have studied whether *α*-MSH has an inhibitory potential on the role of TNF-*α*. We also considered the requirement of melanoma cells to express a wild-type melanocortin receptor for *α*-MSH signalling and whether the *β*1 integrin subunit is a fundamental requirement for migration of melanoma cells.

## MATERIALS AND METHODS

### Human cutaneous melanoma cell culture

The human HBL cutaneous melanoma cell line was originally established in the laboratory of Professor GE Ghanem, University of Brussels, Belgium from a lymph node metastasis of a nodular malignant melanoma (Ghanem *et al*, 1988). Cells were cultured in Ham's F10 medium (Gibco, Paisley, Scotland) supplemented with 5% (v v^−1^) foetal calf serum (FCS), 5% (v v^−1^) neonatal calf serum (NCS) (Sigma, Poole, Dorset, UK), 2 mM L-glutamine, 100 U ml^−1^ penicillin and 100 *μ*g ml^−1^ streptomycin sulphate. The human C8161 melanoma line was established from an abdominal wall metastasis from a menopausal woman with recurrent melanoma (kindly donated by Professor F Meyskens, University of California, Irvine, USA, via Professor M Edwards University of Glasgow, UK). Cells were cultured in Eagle's modified essential medium (EMEM) supplemented with 10% (v v^−1^) FCS, 2 mM L-glutamine, 100 U ml^−1^ penicillin and 100 *μ*g ml^−1^ streptomycin sulphate, 1.2 mg ml^−1^ amphotericin B, 1.5% (v v^−1^) (of a 100 × stock solution) vitamin concentrate, 1 mM sodium pyruvate, 1% (v v^−1^) nonessential amino acids (NEA) and 0.187% (w v^−1^) sodium hydrogen carbonate (Sigma, Poole, Dorset, UK). Cells were incubated at 37°C in a humidified 5% carbon dioxide/95% air environment under standard conditions and passaged prior to confluence using 0.02% (w v^−1^) ethylenediamine tetraacetic acid (EDTA). Cells were used between passages 30–35 for experimentation and were grown to 60% confluence prior to incubation with *α*-MSH (10^−12^–10^−6^ M; Sigma, Poole, Dorset, UK), TNF-*α* (100–750 U ml^−1^; Sigma, Poole, Dorset, UK) or a coincubation of the two as detailed previously (Hedley *et al*, 1998; Haycock *et al*, 1999a,1999b,^2000^). For experiments using a neutralising antibody to the *β*1 integrin subunit, cells were grown under standard conditions to 60% confluence, then incubated with an anti-*β*_1_ integrin neutralising antibody (4 *μ*g ml^−1^) for 24 h (Upstate Biotechnology, Lake Placid, NY, USA).

### Generation of a C8161 human melanoma line stably transfected with the melanocortin-1 receptor

A stable C8161 melanoma cell line expressing functional MC-1 receptor was isolated by transfecting C8161 cells with vector pRc/CMV carrying MC-1 receptor cDNA (kindly donated by Professor JES Wikberg, University of Uppsala, Sweden). Briefly, cells were plated in 100 mm dishes in complete EMEM media. After growth to approximately 70% confluence, cells were transfected with 15 *μ*g of pRC/CMV/MC-1 DNA using Tfx™-50 Reagent, according to the manufacturer's (Promega, UK) protocol (together with a *β*-galactosidase reporter construct). Individual transfectants were isolated and cloned by limiting dilution following growth in complete EMEM media containing 500 *μ*g ml^−1^ geneticin (G-418). Transfection efficiency was confirmed via the *β*-galactosidase-cotransfected reporter gene.

### Assessment of melanoma cell migration using a scratch migration assay: analysis by continuous time-lapse video microscopy

A modified ‘scratch’ migration assay was used for the assessment of the migration of melanoma cells, previously described by Cha *et al* (1996) for studying keratinocyte cell migration. Human melanoma cell line and C8161 cells (which have a doubling time of 24 and 20 h, respectively) were cultured in two 25 cm^2^ sample flasks until 90% confluent, and incubated with TNF-*α* or *α*-MSH (alone or in combination) for 4–32 h. A migration gap of approximately 1 mm was then created by introducing a ‘scratch’ to the adherent layer of cultured cells using a sterile Gilson 1 ml pipette tip. The scratch was administered by hand using a sufficiently applied degree of pressure to remove adherent cells from the polystyrene substrate, but not enough to cause a physical damage to the polystyrene surface. The repeated nature of this task with practice resulted in a cell-free gap of 1.0±0.12 mm (*n*=3) between two adjoining areas of melanoma cells at 90% confluence. At this point, half of the culture medium was removed and replaced with fresh medium to reduce the number of cells introduced into suspension reattaching to the cell-free zone during experimentation. Flasks were then incubated in a 95% air/5% CO_2_ environment for 1 h before being tightly sealed to maintain the above environment during experimentation. Cultures were filmed by video synchronisation (EOS Electronics AV Ltd, UK) using a Leitz DM-IRB inverted microscope (Leica UK Ltd) at 37°C. Migration of the melanoma cells from the two regions of 90% adherent culture density in to the cell-free zone was observed over a 24 h time period and recorded by video microscopy (one frame being recorded every 2 min). Films were then analysed and the number of cells migrating into the cell-free zone created by the scratch of area 1.0±0.12 mm in width by 2.0 mm in length (corresponding exactly with the microscope optic field-of-view) were counted manually at intervals of 4, 8, 12, 16, 20 and 24 h.

### Assessment of melanoma cell migration using a scratch migration assay: analysis by time-interval optical imaging microscopy

The above method was developed further for enabling multiple samples to be analysed during a single experiment (as opposed to two-sample measurements, which proved to be highly accurate but unsuitable for simultaneous study). Cells were cultured as above, but in 24-well plates of 1.5 cm diameter. Prior to cell seeding, the underside of the tissue culture well was marked into six quadrants with an indelible pen. Two linear ‘scratches’ were placed across the adherent monolayer creating cell-free zones as above, ensuring that each scratch traversed all six quadrants in each well. Scratches were examined under a microscope and three were selected for experimentation on the basis of uniformity of area. Micrograph images of the scratch were taken at time=0 min and then TNF-*α* or *α*-MSH (alone or in combination) was added as required. The width of the cell-free zone was then measured at 0, 4, 8 and 24 h after cytokine or peptide addition using Openlab v3.0.2, image analysis software (Improvision, Coventry, UK) and the migrating front of cells was calculated accordingly as a measure of migration speed.

### Statistics

Mean and standard error of the mean values were used for establishing statistical significance between control and cytokine/peptide-incubated samples using Students' paired *t*-test. Significance was taken when *P*⩽0.05. Data are expressed in the Results section as a percentage of control.

## RESULTS

### Migration of cultured HBL and C8161 human melanoma cells

Figure 1

**Figure 1:**
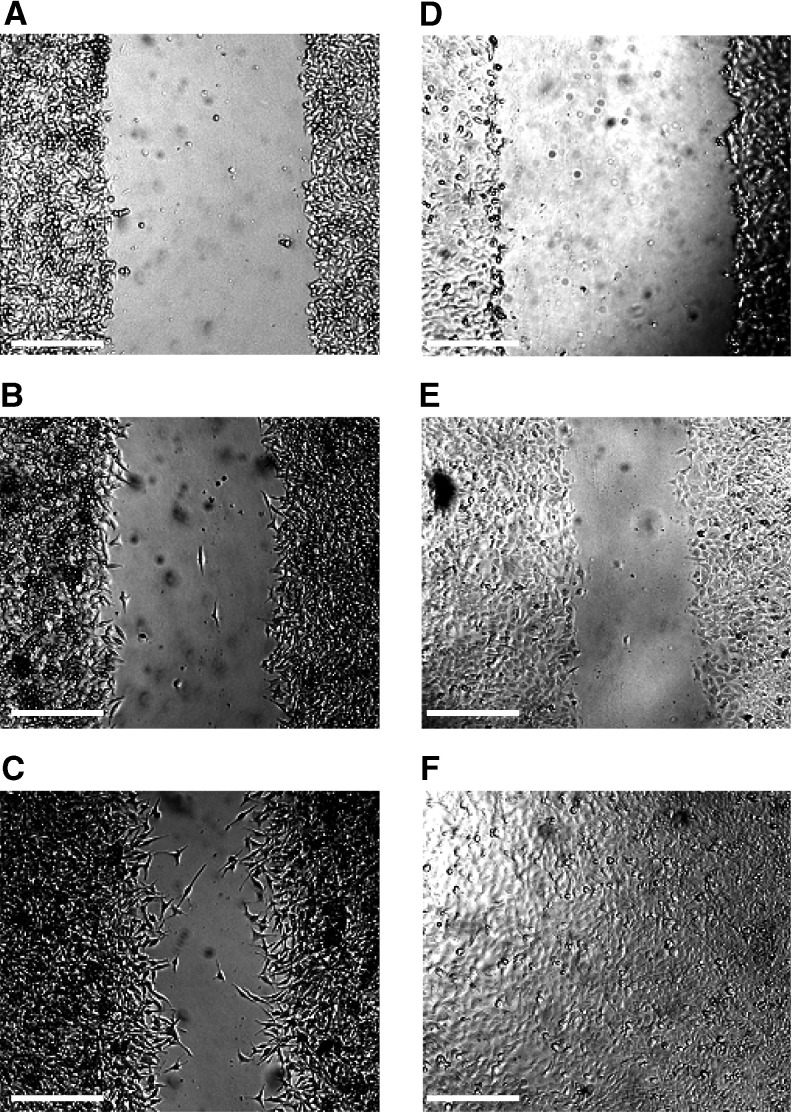
Cultured human HBL and C8161 melanoma cells were investigated for potential to migrate into a cell-free scratch region. This is illustrated for HBL melanoma cells cultured for: (**A**) 0 h; (**B**) 8 h and (**C**) 24 h after administration of the scratch, and similarly for cultured C8161 melanoma cells at: (**D**) 0 h; (**E**) 8 h and (**F**) and 24 h after a scratch was made. Bar=0.5 mm.

shows phase-contrast optical micrographs of the two cultured melanoma cell lines (C8161 and HBL) at 0, 8 and 24 h after creation of a cell-free zone using the ‘scratch’ technique. It can be seen that at time=0, two distinct regions are observed where melanoma cells at 90% confluence are separated by a cell-free zone of approximately a 1 mm width, created by the pipette tip scratch. A continuous rapid and random movement was observed for all cells within minutes, but a resultant movement of a melanoma cell migration front was clearly evident over 24 h, where a highly confluent (90–100%) monolayer region gradually migrated into the cell-free ‘scratch’ region. The C8161 melanoma cells migrated more rapidly than the HBL cells over the 24-h time period, due to a faster migration speed (Figures 1 and 2A

**Figure 2:**
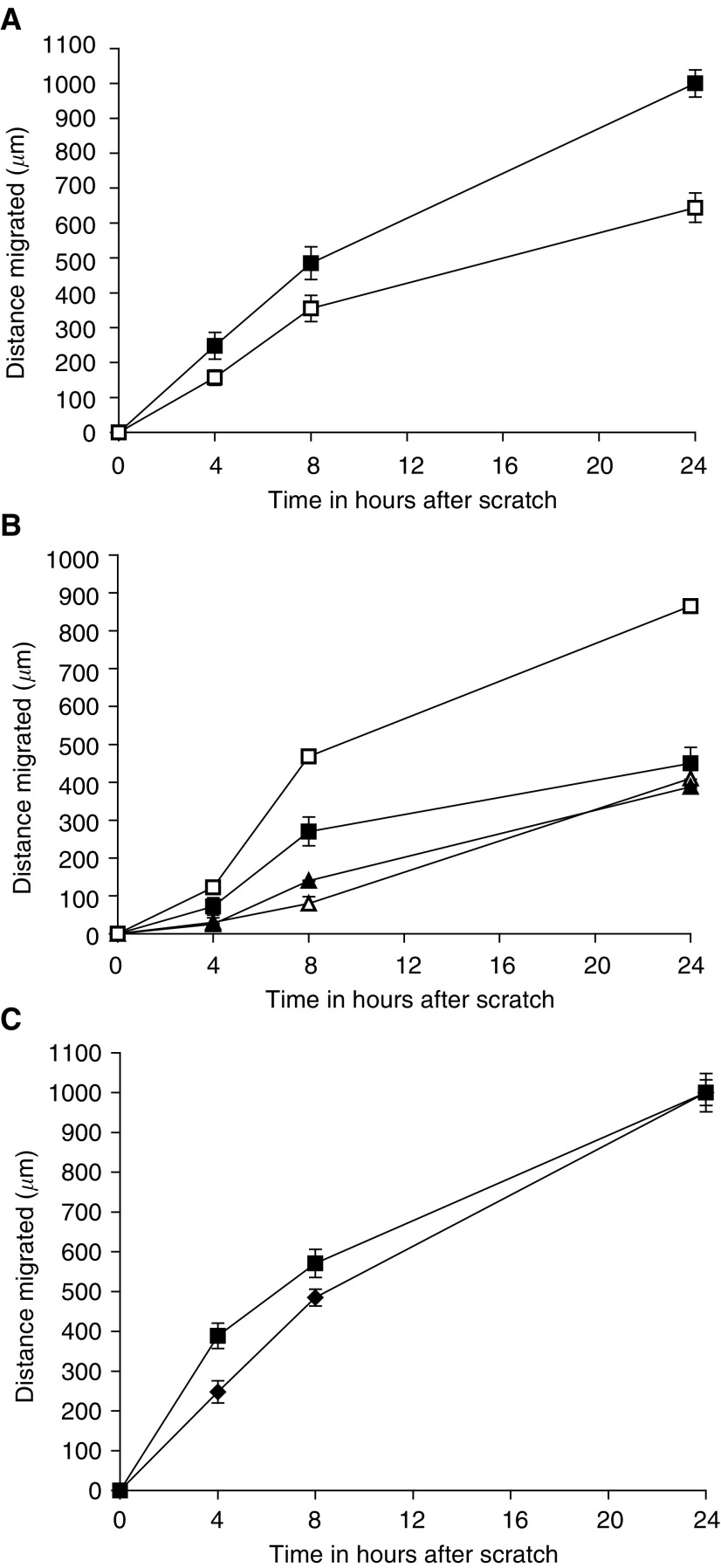
(**A**) Distance migrated of cultured HBL (□) and C8161 (▪) melanoma cells over 24 h using the scratch assay technique. (**B**) Time course of TNF-*α* (300 U ml^−1^) action on HBL melanoma cell migration. Key: (▴) no TNF-*α*; (▵) 4 h preincubation with TNF-*α*; (▪) 8 h preincubation with TNF-*α* and (□) 24 h preincubation with TNF-*α*. (**C**) The effect of TNF-*α* (300 U ml^−1^) on migration of C8161 melanoma cells. Key: (⧫) no TNF-*α* and (▪) cells pretreated with TNF-*α* for 24 h prior to administration of scratch. Values shown are mean±s.e.m. (*n*=3).

). It is important to note that the C8161 line has a 20-h doubling time and the HBL line a 24-h doubling time. However, from visual analysis of the time-lapse microscopy, it is clearly evident that occupation of the gap is predominantly via migration, rather than a simple doubling of the cells (data present as a separate avi file). A comparison of the two analytical techniques for assessing migration revealed that similar data was obtained irrespective of whether time-lapse video microscopy or time-interval image analysis was used. Therefore, subsequent experiments were conducted using time-interval image analysis. Data is described as the migration distance attained, measured by a reduction in the width of the initial scratch.

### Tumour necrosis factor *α* increases migration of HBL and C8161 melanoma cells

**Figure 3:**
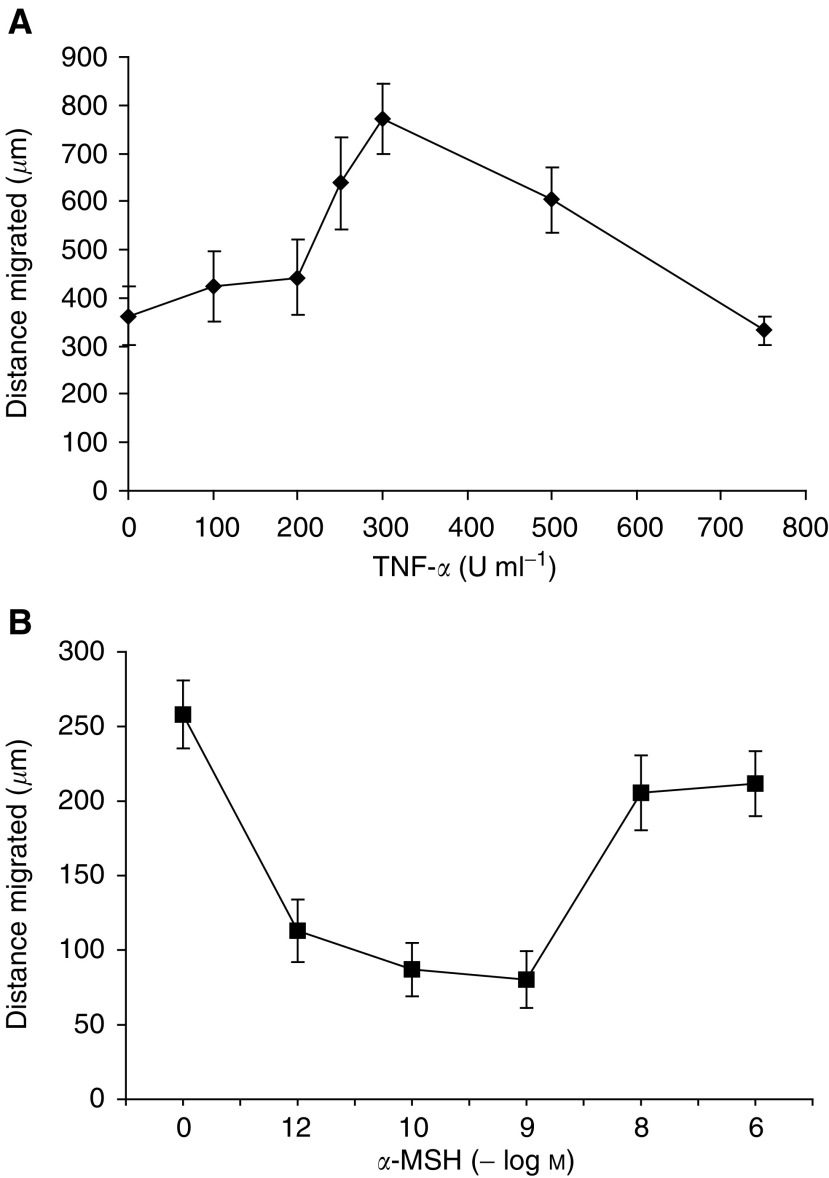
(**A**) The dose-dependant effect of TNF-*α* on the migration of HBL melanoma cells over a 24 h time period (cells were preincubated with TNF-*α* for 24 h prior to scratch administration). (**B**) The dose-dependant effect of *α*-MSH on inhibiting the migration of HBL melanoma cells stimulated with TNF-*α* (300 U ml^−1^) over 24 h. Values shown are mean±s.e.m. (*n*=3).

Preincubation of HBL and C8161 melanoma cells with TNF-*α* (300 U ml^−1^) prior to introducing a ‘scratch’ resulted in an increase in the rate of migration of both melanoma cell lines (Figure 2). For HBL cells, the greatest increase in migration speed was observed when cells were preincubated for 24 h (Figure 2B). Preincubation for 4 h had no effect on migration speed, and preincubation for 8 h revealed a faster migration than control cells, but not as fast as a 24 h incubation (Figure 2B). A similar time course of action of TNF-*α* preincubation was observed with C8161 cells (Figure 2C). However, as these cells displayed a faster basal migration speed compared to HBL cells, we found that after 24 h no difference was observed between TNF-*α*-stimulated and unstimulated cells. This was due to control cells migrating completely in the assay system. The greatest increase in migration speed for HBL cells was observed when TNF-*α* at 300 U ml^−1^ was used with a preincubation time of 24 h (Figure 3A

). Time-lapse video microscopy data for TNF-*α*-stimulated C8161 cells is present as an avi file.

### *α*-Melanocyte-stimulating hormone inhibits TNF-*α-*stimulated migration of HBL melanoma cells but not C8161 melanoma cells

**Figure 4:**
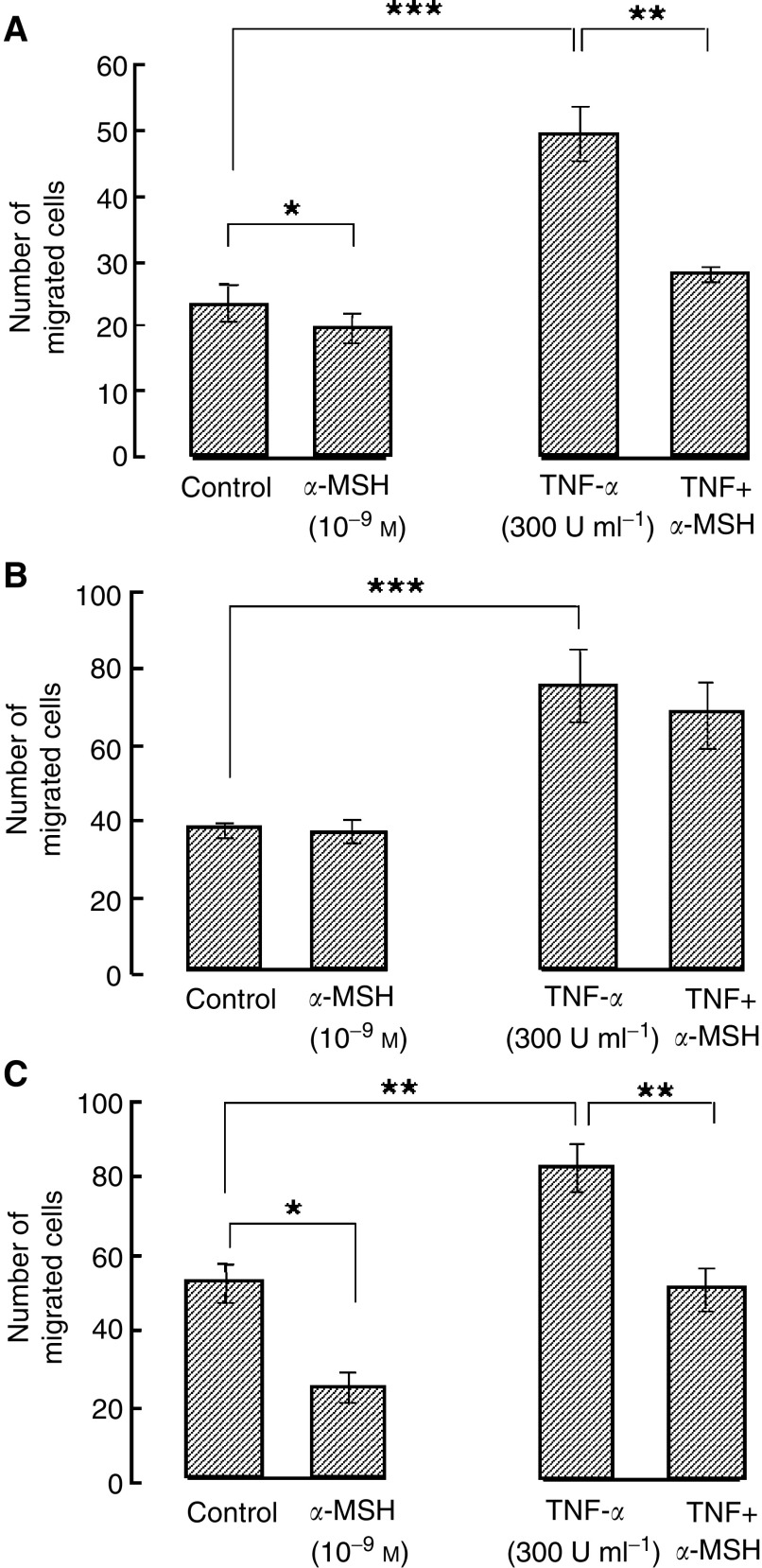
(**A**) The comparative effect of *α*-MSH (10^−9^ M) on control unstimulated and TNF-*α*-stimulated (300 U ml^−1^) migration of cultured HBL melanoma cells, (**B**) C8161 melanoma cells and (**C**) C8161 melanoma cells stably transfected with the melanocortin-1 receptor. Values shown are mean±s.e.m. (*n*=3). ^*^P<0.05, ^**^P<0.01, ^***^P<0.001.

The above experiments investigating the preincubation time and the concentrations of TNF-*α*-stimulating melanoma cell migration enabled us to select a 24 h preincubation of TNF-*α* at 300 U ml^−1^ and a migration time point of 24 h for investigating the action of *α*-MSH on melanoma migration. *α*-Melanocyte-stimulating hormone at a concentration range of 10^−12^–10^−9^ M had a significant inhibitory effect on HBL cell migration, whereas higher concentrations of 10^−8^–10^−6^ M did not (Figure 3B). Figure 4

compares the response to *α*-MSH (10^−9^ M) on unstimulated and TNF-*α* (300 U ml^−1^)-stimulated HBL and C8161 cells. In HBL cells (Figure 4A), TNF-*α* (300 U ml^−1^) significantly increased cell migration (*P*<0.001), while *α*-MSH (10^−9^ M) significantly decreased HBL migration alone (*P*<0.05) and in combination with TNF-*α* (*P*<0.01). In contrast, Figure 4B illustrates that while TNF-*α* (300 U ml^−1^) alone significantly increased migration of C8161 cells (*P*<0.001), *α*-MSH failed to influence unstimulated or TNF-*α*-stimulated migration for these cells. However, when C8161 cells were transfected with the wild-type melanocortin-1 receptor (Figure 4C), *α*-MSH at 10^−9^ M significantly reduced unstimulated C8161 migration (*P*<0.05) and also completely blocked the response of the transfected cells to TNF-*α* (*P*<0.01). The procedure of transfecting C8161 cells was not found to influence the absolute rate of migration significantly compared with untransfected cells, as can be seen by comparing both control and TNF-*α*-stimulated and migration rates between these two populations of cells (Figure 4B and C).

### Neutralising antibody to *β*_1_ integrin inhibits the migration of HBL and C8161 melanoma cells

**Figure 5:**
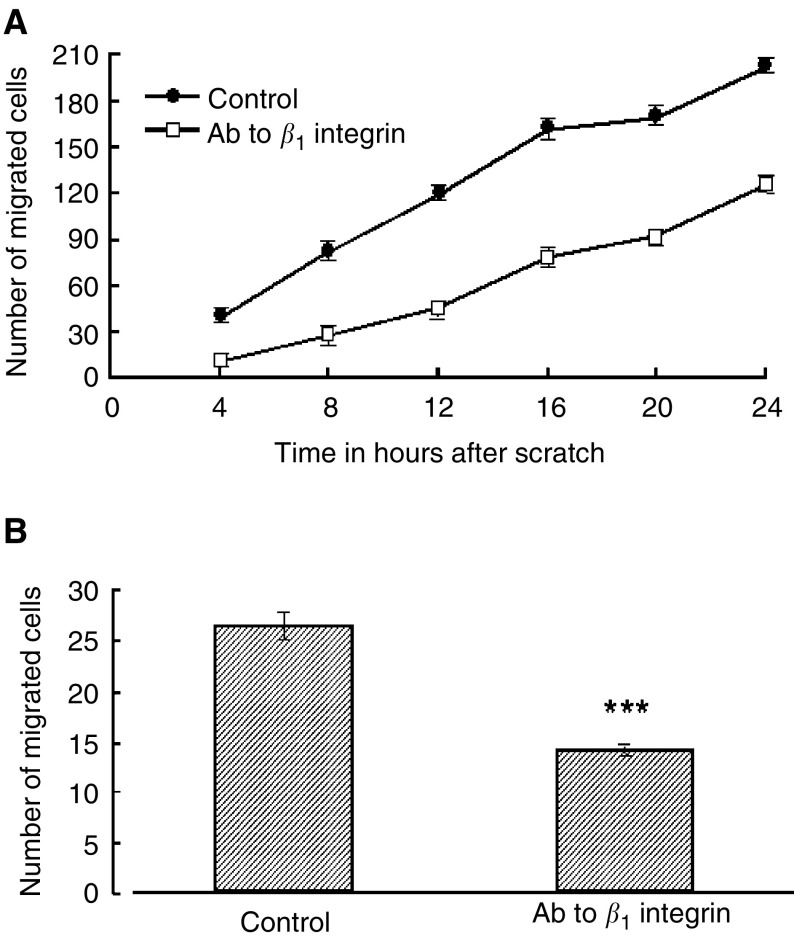
(**A**) The effect of a neutralising antibody to the *β*_1_ integrin subunit on migration of C8161 melanoma cells over 24 h following administration of a scratch. Key: (•) control, no antibody and (□) anti-*β*1 integrin antibody (4 *μ*g ml^−1^). (**B**) Effect of neutralising antibody to *β*_1_ integrin subunit on migration of HBL melanoma cells at 24 h after scratch. Values shown are mean±s.e.m. (*n*=3). ^*^P<0.05, ^**^P<0.01, ^***^P<0.001.

The addition of a neutralising antibody to melanoma cells was observed to significantly inhibit their ability to migrate over a 24-h time period. Data for the C8161 cells are given in Figure 5A

and for HBL cell in Figure 5B. Addition of neutralising antibody to the integrin *β*_1_ subunit reduced the rate of migration of HBL and C8161 cells by 46±3.2 and 48±2.8%, respectively, 24 h after the formation of a scratch. Time-lapse video microscopy data for C8161 cells plus anti-integrin *β*1 is present as an avi file. Control experiments using an isotype IgG control antibody did not significantly affect the migration speed of either cell type.

## DISCUSSION

The aim of this study was to investigate the effect of the proinflammatory cytokine TNF-*α* and the anti-inflammatory peptide *α*-MSH on melanoma cell migration. This work extends recently published findings from our group describing TNF-*α* increased melanoma cell attachment, invasion through fibronectin and expression of integrins *α*3, *α*4 and *β*1 (Zhu *et al*, 2002). The present study demonstrates that melanoma cell migration is increased by TNF-*α* in two melanoma cell lines. We also show that the HBL cell line (which has a wild-type melanocortin-1 receptor) responds to *α*-MSH with a reduction in TNF-*α*-stimulated migration. In contrast, the C8161 cell line (which has a polymorphic loss of function of the MC-1 receptor) does not respond to the action of *α*-MSH. However, when these cells are stably transfected with the wild-type MC-1 receptor, it restores *α*-MSH function, such that the cells then responded with a significant reduction in TNF-*α*-stimulated migration.

Proinflammatory cytokines TNF-*α* and IL-1*α* can upregulate *α*4, *α*5 and *α*6-melanoma integrin expression, with an associated increase in migration on fibronectin (Giavazzi *et al*, 1990; Dekker *et al*, 1994). In support of this, we have reported previously that human HBL melanoma cells in culture respond to TNF-*α* with an upregulation of *α*3, *α*4 and *β*1 integrin expression (Zhu *et al*, 2002). This was associated with an increase in fibronectin invasion. In addition, the anti-inflammatory peptide *α*-MSH was effective at decreasing HBL cell attachment and invasion (Zhu *et al*, 2002).

The *in vivo* invasion of melanoma cells probably involves two components – an increase in the rate of cellular migration and an increase in the proteolytic activity necessary to degrade the extracellular matrix. The current *in vitro* study investigated the extent to which the stimulatory action of TNF-*α* on melanoma invasion is explained by an action on cellular migration rather than on proteolytic degradation of the surrounding matrix. *In vitro* and *in vivo* invasion may involve both increased migration and increased proteolytic breakdown of the matrix. Thus, we recently found that while TNF-*α* did not cause an obvious upregulation of proteolytic activity in HBL cells, the introduction of a broad-spectrum protease inhibitor (*α*_2_ macroglobulin) was able to block TNF-*α*-stimulated cell invasion and cell migration (Katerinaki *et al*, 2003). In the current study, we compared two methods for measuring cell migration, both based on introducing a ‘scratch’ to a cultured monolayer of cells (adapted from a method previously reported for keratinocytes (Cha *et al*, 1996)). One method used time-lapse video microscopy and counted the number of cells entering a defined cell-free area in a given period of time. The other relied on a computer software image analysis measurement of the decreasing width of the remaining scratch as the melanoma cell ‘fronts’ advanced. The video microscopy approach had a technical advantage of enabling a complete experiment to be run without physical disturbance (as incubator conditions were present during recording). This was useful for experiments conducted using cells that have a relatively weak affinity for the surface substratum (such as the HBL melanoma cell line). A second major advantage of this method is that it allows the continuous viewing of all cells, enabling one to look at whether changes in migration velocity were attributable equally to all cells, to a subpopulation of cells or was due predominantly to the rate of cell doubling (as opposed to migration). This did not appear to be the case in these experiments. However, cell migration using this method was quantified by manually counting cells (which is a tedious and time consuming process). Another major experimental limitation was that we were only able to process two samples at a time, limiting the number of variables investigated in any one experiment.

In contrast, the image analysis method of the multiple scratches per well allowed replicate data points and dose–response experiments to be undertaken. It did require cultured cells to be physically moved between tissue culture incubator and microscope for assessment, thus care had to be taken when handling weakly adherent cells. This problem is minimised by avoiding complete medium changes (which can detach all attached cells) and careful physical handling. Both methods of assessment relied upon scratches (using a polystyrene disposable pipette tip) that needed to be as reproducible as possible in width. In practice, we found that it was feasible to produce scratches of a reproducible width by conducting sufficient replicates. These could be run so that irregular scratches were identified and discarded prior to entry into experiments. Similar approaches using this methodology have been useful in determining the migration speed of ovarian carcinoma cells, showing that the *α*-2 integrin subunit is essential for migration (Kokenyesi *et al*, 2003). The role of retinoic acid at inhibiting the migration of neuroblastoma cells has also been described using this technique (Voigt and Zintl, 2003).

Similar results were obtained for both HBL and C8161 cells irrespective of the method of assessment. Both cells were highly responsive to TNF-*α* and the time course of action (18–28 h) was consistent with this cytokine causing an upregulation of integrin subunits, which could be responsible for an increase in migration (Zhu *et al*, 2002). The inhibitory effect of a neutralising antibody to the *β*_1_ integrin subunit was consistent with this. The finding that TNF-*α* increases the migration of melanoma cells is consistent with and strongly supports an inflammatory environment promoting melanoma metastases. In addition, *α*-MSH blocked the response to TNF-*α* for the HBL melanoma cells but not the C8161 line, and is therefore consistent with an anti-inflammatory action of this molecule.

Melanocortin peptides interact with a family of melanocortin receptors, MC-1R to MC-5R. The HBL and C8161 cells both possess melanocortin type 1 and 2 receptors (Eves *et al*, 2003, in press). However, the HBL cell line is wild type for MC-1R, while the C8161 cell has a nonfunctional variant (Sanchez-Mas *et al*, 2002). In this study, the HBL cells responded to *α*-MSH with a reduction in migration, whereas the C8161 cells did not. However, when C8161 cells were stably transfected with the wild-type MC-1 receptor, they responded to *α*-MSH with a significant reduction in cell migration. Stable transfection of the C8161 melanoma cells with the wild-type MC-1R and the restoration of an *α*-MSH response provides strong evidence that this peptide is acting by the MC-1R receptor to reduce cell migration. The dose–response curve to *α*-MSH showed that relatively low concentrations were capable of opposing TNF-*α*, whereas higher concentrations were without effect. Our own group (Morandini *et al*, 1998; Hedley *et al*, 1998,^2000^; Haycock *et al*, 1999a,1999b,^2000^; Moustafa *et al*, 2002) and others (Lipton and Catania, 1997) have shown previously that the anti-inflammatory and anti-oxidative properties of *α*-MSH often seem to be more effective at lower rather than higher concentrations. It is not entirely known why this is observed, but we suspect that dual signalling pathways from the melanocortin receptor account for this. A long-standing problem in precisely dissecting the mechanism of *α*-MSH action is the lack of neutralising antibodies to the MC-1R receptor or a functional antagonist for *α*-MSH.

In conclusion, we provide evidence that TNF-*α* has a dramatic effect on migration of human cutaneous melanoma cells *in vitro* and that *α*-MSH plays an important role in reducing cell migration and opposing the promigratory effects of TNF-*α*. Furthermore, we substantiate that the inhibitory action of *α*-MSH requires the expression of a functional MC-1 receptor. We also show that the *β*_1_ integrin subunit is required for melanoma cell migration. The study also provides a simple model for following cell migration, which can be used to investigate new pharmaceutical approaches for investigating melanoma invasion/migration.
